# Stable feature selection based on the ensemble *L*_*1*_-norm support vector machine for biomarker discovery

**DOI:** 10.1186/s12864-016-3320-z

**Published:** 2016-12-22

**Authors:** Myungjin Moon, Kenta Nakai

**Affiliations:** 1 Department of Computational Biology and Medical Sciences, Graduate school of Frontier Sciences, The University of Tokyo, 5-1-5 Kashiwanoha, Kashiwa-shi, Chiba-ken 277-8562 Japan; 2 Human Genome Center, The Institute of Medical Science, The University of Tokyo, 4-6-1 Shirokanedai, Minato-ku, Tokyo, 108-8639 Japan

## Abstract

**Background:**

Lately, biomarker discovery has become one of the most significant research issues in the biomedical field. Owing to the presence of high-throughput technologies, genomic data, such as microarray data and RNA-seq, have become widely available. Many kinds of feature selection techniques have been applied to retrieve significant biomarkers from these kinds of data. However, they tend to be noisy with high-dimensional features and consist of a small number of samples; thus, conventional feature selection approaches might be problematic in terms of reproducibility.

**Results:**

In this article, we propose a stable feature selection method for high-dimensional datasets. We apply an ensemble *L*_*1*_-norm support vector machine to efficiently reduce irrelevant features, considering the stability of features. We define the stability score for each feature by aggregating the ensemble results, and utilize backward feature elimination on a purified feature set based on this score; therefore, it is possible to acquire an optimal set of features for performance without the need to set a specific threshold. The proposed methodology is evaluated by classifying the binary stage of renal clear cell carcinoma with RNA-seq data.

**Conclusion:**

A comparison with established algorithms, i.e., a fast correlation-based filter, random forest, and an ensemble version of an *L*_*2*_-norm support vector machine-based recursive feature elimination, enabled us to prove the superior performance of our method in terms of classification as well as stability in general. It is also shown that the proposed approach performs moderately on high-dimensional datasets consisting of a very large number of features and a smaller number of samples. The proposed approach is expected to be applicable to many other researches aimed at biomarker discovery.

## Background

Biomarker discovery has become one of the most significant research objectives in recent years. Because biomarker discovery is typically modeled to determine the most discriminating features for classification, it can be described as a feature selection problem regarding class from the point of view of machine learning [1–3]. Feature selection is the step that involves identification of the most salient features for learning [4] and enables the performance of the classifier to be enhanced by eliminating irrelevant features that cause inaccurate prediction or over-fitting problems. In addition, the time required for learning is reduced as feature selection serves to lower the dimensionality. Although classification without a feature selection process may improve the classification performance, a large number of features would complicate interpretation of the result. In short, feature selection not only enhances the classification performance, it also improves the understanding and analysis of the data. The emergence of new high-throughput technologies has made genomic data, such as microarray data and RNA-seq, widely available for biomarker discovery. However, the distinct characteristics of biomedical data, which often contain far more features than the number of samples, mean that conventional feature selection approaches might be problematic, especially with regard to reproducibility; small changes in the dataset could lead to large changes in the feature selection result, which should not be considered as important biomarkers. Feature selection in the biomedical field should consider the stability of features, as well as the influence on the classification performance. Some researchers have suggested the ensemble feature selection based on instance perturbation to address this problem [5–7]. This study proved that the stability of selected features was significantly improved by performing feature selection on slightly different datasets and aggregating their results. Furthermore, research that combines lasso regression with resampling was introduced [8, 9]. The *L*_*1*_-norm of lasso regression tends to force the solution to be sparse, and it shows high efficiency for feature selection in regression problems.

In this paper, we propose a stable feature selection method based on the *L*_*1*_-norm support vector machine (SVM). The basic concept of *L*_*1*_-norm SVM is similar to that of lasso regression, but it is tailored for classification tasks, which is the model for many biomarker discoveries. *L*_*1*_-norm SVM efficiently reduces the number of irrelevant or redundant features to fewer than the number of samples; thus, it is appropriate for biomedical high-dimensional data. As our methods are applied over instance perturbation steps, the stability issue, which is one of the most critical problems of *L*_*1*_-norm can also be managed. Furthermore, the optimal subset of selected features was detected by applying backward feature elimination to our own ranking criteria. By eliminating features one by one based on our ranking criteria generated by the *L*_*1*_-norm SVM, a cross-validated classification score is calculated, and a subset of features that maximizes classifier performance is acquired.

The proposed method is tested for the classification of renal clear cell carcinoma stage classification. We used an RNA-seq gene expression dataset of renal clear cell carcinoma samples from the cancer genome atlas (TCGA) for our study. We compared our approach with three established feature selection methods, namely a fast correlation-based filter (FCBF) [10], random forest [11], and an ensemble version of support vector machine-based recursive feature elimination (SVM-RFE) [6, 12], from the point of view of classification performance and the stability of selected features. The experiment showed that our method has considerable classification performance and stability.

## Results

### Datasets

The RNA-seq gene expression data of renal clear cell carcinoma was obtained from Broad GDAC Firehose, which is one of the genome data analysis centers of the TCGA project [13]. Level 3 RNAseqV2 datasets of kidney renal clear cell carcinoma (KIRC) was used for experiments. We only took into account tumor-matched normal samples. The vectors of RNA-seq by expectation maximization (RSEM) [14] that are normalized by z-score were used as an estimate for the gene expression level. We discarded genes and samples that contain invalid or null values. The pathogenic stage information of renal clear cell carcinoma samples is retrieved from TCGA clinical dataset biotab files and set as the class label of gene expression data. Basically the stage is divided into four stages, i.e., stages I, II, III, and IV by TNM stage groupings, which take into account the size of the tumor, the lymph nodes involved and distant metastasis [15]. We took into account only two stages, i.e., stage I and stage IV, as renal clear cell carcinoma of stage I involves local tumors that only exist in the kidney, whereas tumors at stage IV have grown into other tissues outside the kidney or have spread widely to other lymph nodes; thus, the use of these stages could be a significant clue for tumor advance and tumor metastasis. Samples of which the stage information is not clear were not included in the test. After the filtering steps, our dataset consisted of 352 samples, of which 268 and 84 were stage I and stage IV samples, respectively. Each sample consists of the RSEM vector for 20199 genes.

Although cross-validation is one of the most popular methods for classification tests on a biological dataset, it tends to produce a dataset-dependent result especially with a small sample size [16]. Moreover, as the resampling step is part of the compared and proposed approach, a classification test on cross-validation might not hold significant meaning. Hence, the classification performance should be evaluated by an independent data test. We ensured that the test remained independent of the dataset by randomly divided the original dataset into a training set (80%) and a test set (20%). The training set consisted of 214 and 67 stage I and stage IV samples, respectively, whereas the test set contained 54 and 17 samples, respectively. Only the training dataset was utilized for the cross-validation test. The stability test was performed by randomly generating 20 subsets from the original dataset, each of which contained 80% of the whole samples. The FCBF feature selection test was implemented by using weka 3.7.13 [17], and the other experiments were all implemented with Python, using the library scikit-learn 0.17 [18].

### Feature selection

Data perturbation was achieved by producing 1000 bootstrap samples containing 80% of the data from the training set. Then feature selection was performed by first calculating the regularization parameter *C* of *L*_*1*_-norm SVM by grid search, using 10-fold cross-validation on each bootstrap sample of training data. We determined the best value in the range of *C*∈{10^−5^, 10^−4^, …, 10^3^, 10^4^}. Because our dataset contains bias in class proportions, simply considering accuracy as a performance estimator is not appropriate. Instead, we regard the area under the curve (AUC) as the main criterion for performing experiments. Thus, the value of *C* resulting in the best AUC score was selected for each bootstrap sample.

Then *L*_*1*_-norm SVM was applied to 1000 bootstrap samples to filter those genes whose coefficients are 0. We calculate and record the 10-fold cross-validation score of reduced feature sets at this point to choose optimal feature sets for each bootstrap in the later step. These steps are repeated several times until no more feature reduction is available. The remaining genes were aggregated to one gene set and ranked by the number of bootstrap samples that finally remained.

Subsequently, as a revising step, backward feature elimination is performed to find the optimal feature subset of which the classification performance is the best. Again, we used the AUC score as the main criterion for the classification performance. SVM is selected as a classifier, and 10-fold cross-validation is applied for the test. The grid-search method is also applied to set the regularization parameter *C* and *γ* in the range of *C*∈{10^−5^, 10^−4^, …, 10^9^, 10^10^} and *γ*∈{10^−9^, 10^−8^, …, 10^2^, 10^3^}, respectively.

**Fig. 1:**
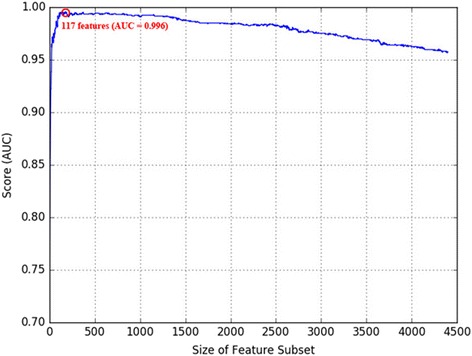
Backward feature elimination for optimal subset. Backward feature elimination is performed based on our ranking criteria. SVM with an RBF kernel is used as a classifier for calculating the cross-validation score. The X- and Y-axes denote the size of the feature subset and the 10-fold cross-validation AUC score, respectively. The red circle indicates the number of features with the highest AUC score in our experiment, which is 177 features with AUC = 0.996

Then the mean AUC score obtained from the cross-validation test is calculated. The score is recursively calculated by reducing the genes one by one, starting from the full gene sets, until a subset consisting of only one gene is tested. Figure 1 demonstrates the alteration of the mean AUC score by the backward feature elimination.

The performance of our model was compared with three well-known feature selection methods, i.e., FCBF, random forest, and an ensemble version of SVM-RFE. FCBF is a feature selection method that is used to remove irrelevant features based on symmetric uncertainty. Although the stability was not considered in FCBF, we selected it as a comparison target because this feature selection algorithm was adapted from a previous study that classified stage progression of renal clear cell carcinoma based on the RNA-seq dataset [19]. Random forest is a method based on decision trees, and frequently has been used for both feature selection and classification. Random forest deals with the stability issue by bootstrap aggregation (bagging) included in the algorithm. SVM-RFE is a feature selection method that recursively eliminates the features whose weight magnitudes of *L*_*2*_-norm SVM are the smallest [12], and it has been proven to deliver superior performance in many recent studies. However, SVM-RFE is problematic in terms of stability when applied to a single dataset; thus, some previous researchers have tried to use the ensemble version of SVM-RFE based on instance perturbation to address this problem. [6, 20] For the ensemble SVM-RFE in our comparison experiment, the fraction ratio for elimination in each step was set to 20%, and a linear aggregation method that sums the rank over all bootstraps was used as [6]. The number of bootstraps was set to 1000, and regularization parameter *C* is optimized in the same way as our method. As random forest and SVM-RFE provides only the ranking list of all features, we applied backward feature elimination method to find the optimal subset of features, similar to our method. The 10-fold cross-validation score is calculated by the classifier while adding features one by one, based on the feature rankings. Then the optimal feature set which maximizes the cross-validation score is acquired. The random forest classifier and SVM classifier with the radial basis function (RBF) kernel were used as classifiers for the random forest and the ensemble SVM-RFE, respectively.

### Classification performance test

**Fig. 2:**
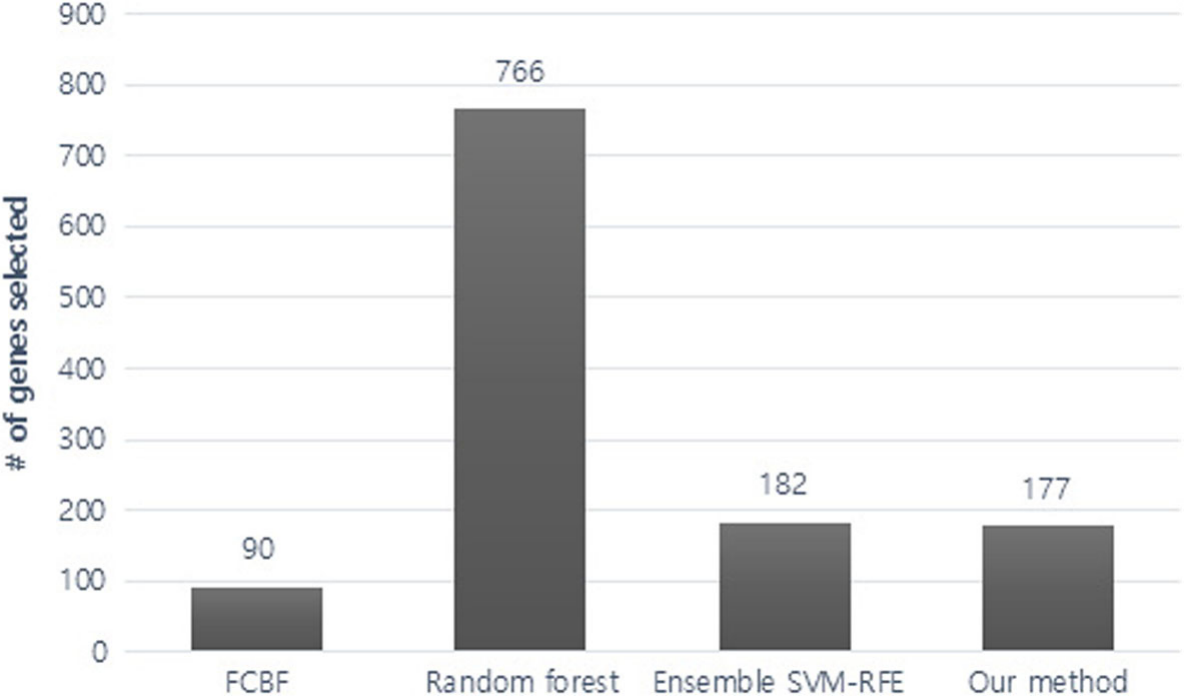
Number of genes selected by each method. The figure shows the number of genes selected by each feature selection method, i.e., FCBF, random forest, ensemble SVM-RFE and our method

A cross-validation test as well as an independent dataset test is conducted for evaluation of classification performance. As described in the subsection on datasets, the data that was included in the independent test set was not used for the feature selection process at all. Only the training set was used for the cross-validation test. Four popular classification methods, i.e., adaptive boosting (AdaBoost), logistic regression, random forest, and SVM with an RBF kernel were used for performance evaluation. Gene selection was performed by FCBF, random forest, ensemble SVM-RFE, and our method before the classification tests. Figure 2 demonstrates the number of genes and selected by each method. As random forest selects far more genes than other methods, an additional test with 180 genes is conducted using random forest, which is similar to the number of genes we used in the ensemble SVM-RFE method and our method. Then we assessed four performance measures, i.e., the accuracy, f1 score, Matthews correlation coefficient (MCC), and area under the curve (AUC) for each classifier. Tables 1 and 2 compare the classification performance of the approaches for the independent data test and cross-validation test, respectively. As seen in the tables, our algorithm shows the overall best performance for most classifiers among most performance indices, both in the cross-validation test and independent dataset test. Especially, our method with the SVM classifier demonstrated the best performance among all.

**Table 1:** Classification performance test with the independent dataset

Classifier	Performance measure	FCBF	Random forest (766)	Random forest (180)	Ensemble SVM-RFE	Our method
AdaBoost	Accuracy	0.789	0.845	0.859	0.887	**0.901**
	F1-score	0.545	0.593	0.667	0.75	**0.774**
	MCC	0.408	0.532	0.588	0.68	**0.717**
	AUC	0.7	0.717	0.766	0.825	**0.834**
Logistic regression	Accuracy	0.789	0.789	0.817	**0.845**	**0.845**
	F1-score	0.651	0.595	0.667	0.718	**0.732**
	MCC	0.533	0.456	0.552	0.623	**0.646**
	AUC	0.801	0.74	0.799	0.838	**0.858**
Random forest	Accuracy	0.817	0.817	0.803	0.831	**0.845**
	F1-score	0.48	0.48	0.462	0.5	**0.56**
	MCC	0.426	0.426	0.381	0.479	**0.531**
	AUC	0.658	0.658	0.649	0.667	**0.697**
SVM	Accuracy	0.803	0.831	0.859	0.831	**0.901**
	F1-score	0.632	0.571	0.583	0.684	**0.811**
	MCC	0.504	0.489	0.589	0.577	**0.749**
	AUC	0.77	0.708	0.706	0.808	**0.895**

The performance score is calculated on the independent dataset. The items that obtained the best score are highlighted in bold text. The numbers below the random forest classifier denote the number of genes selected for the performance test

**Table 2:** Classification performance test with cross-validation

Classifier	Performance measure	FCBF	Random forest (766)	Random forest (180)	Ensemble SVM-RFE	Our method
AdaBoost	Accuracy	0.872	0.882	0.85	0.886	**0.889**
	F1-score	0.737	**0.749**	0.647	0.738	0.735
	MCC	0.668	0.677	0.568	0.676	**0.686**
	AUC	0.902	0.923	0.868	0.936	**0.944**
Logistic regression	Accuracy	0.833	0.853	0.822	0.957	**0.978**
	F1-score	0.704	0.722	0.664	0.915	**0.958**
	MCC	0.609	0.636	0.566	0.894	**0.947**
	AUC	0.904	0.893	0.853	0.994	**0.997**
Random forest	Accuracy	**0.871**	0.84	0.844	0.83	0.833
	F1-score	0.614	0.553	**0.625**	0.459	0.45
	MCC	**0.579**	0.504	0.557	0.473	0.457
	AUC	0.918	0.869	0.851	0.924	**0.928**
SVM	Accuracy	0.879	0.854	0.84	0.95	**0.968**
	F1-score	0.762	0.659	0.589	0.895	**0.933**
	MCC	0.692	0.58	0.514	0.865	**0.914**
	AUC	0.915	0.885	0.871	0.992	**0.996**

The mean performance score of 10-fold cross validation test is calculated. The items that obtained the best score are highlighted in bold text. The numbers below the random forest classifier denote the number of genes selected for the performance test

### Stability test

**Fig. 3:**
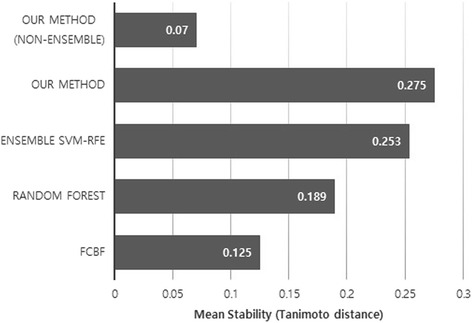
Mean Tanimoto distance. The arithmetic mean of the Tanimoto distance on 20 random subsamples is calculated as a stability score. The X-axis denotes the arithmetic mean of the Tanimoto distance of each method

**Fig. 4:**
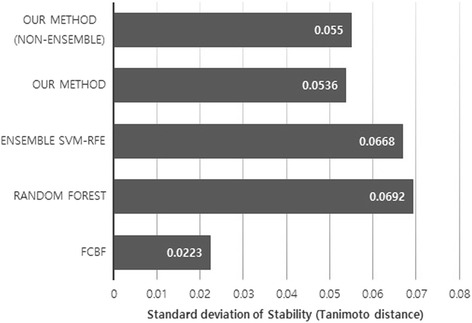
Standard deviation of Tanimoto distance. The standard deviation of the Tanimoto distance on 20 random subsamples is calculated. The X-axis denotes the standard deviation of the Tanimoto distance of each method

We carried out the stability test by creating 20 random subsamples from the original dataset, which is constructed with 80% of the data. The stability test was performed by using the Tanimoto distance *T*. Here, we also tested our method without bagging for the comparison. Figures 3 and 4 demonstrate the mean and standard deviation of the Tanimoto distance for each method, respectively. As we see in the figures, our method generally demonstrates high stability among all the methods. The ensemble SVM-RFE shows the similar performance as our method. However, *L*_*1*_-norm SVM without instance perturbation shows remarkably lower scores than other methods, which proves the significance of the ensemble selection for *L*_*1*_-norm-based methods. FCBF also displays a relatively lower stability score than other methods as it does not take into account the stability issue, although it shows the least variance, since it selects almost the same number of features within subsamples.

## Discussion and Conclusion

In this paper, we present a novel feature selection method based on *L*_*1*_-norm SVM over data perturbation efficient for biomarker discovery. The nature of the *L*_*1*_-norm that leads to a sparse solution provides a fairly efficient way for feature selection for high-dimensional data. *L*_*1*_-norm SVM is also suitable for biomarker selection, as it delivers high performance in classification, and is applicable to diverse situations. However, the use of *L*_*1*_-norm SVM on a single dataset has difficulty in detecting closely correlated factors, which is common in biomarker detecting. In addition, it may produce a result that is subordinate to a certain dataset. In our experiments, the stability of features could be improved as *L*_*1*_-norm SVM is applied to a number of bootstrap samples, considering instance perturbation. Instead of using the general ranking criteria of SVM, we consider only the number of bootstrap samples that selected the feature as measure of the stability. By applying backward feature elimination based on our own stability score, we could determine an optimal subset of features that holds good performance for classification. We applied our approach to RNA-seq data of renal clear cell carcinoma to find candidate biomarkers related to stage progress, which might be closely associated with tumor advance and the metastasis issue. Through comparison with established feature selection methods, the performance of our algorithm was proved in terms of classification performance and stability.

The stability of feature selection is a significant issue and its importance has been underestimated for a long time; yet, many research efforts aimed at feature selection solely focused on the performance of the methods. However, as we can see from the case of non-ensemble methods in our experiment, feature selection algorithms designed without considering stability might find many different subsets of features, if the data changes even slightly. This causes low reproducibility in high-dimensional datasets, such as microarray data or RNA-seq, and would make the result of the analysis less meaningful. Thus, stable feature selection is an essential issue in biomarker discovery. Of course, the performance of features should not be neglected because feature stability does not guarantee true biomarker detection.

Although it is based on a simple idea, the proposed approach was moderately successful on datasets consisting of a very large number of features and relatively much smaller samples. Our research proposes a general process for binary classification problems on high-dimensional data and we expect the proposed method to be applicable to many other kinds of biomarker discovery. However, although it generally demonstrated improved performance compared to conventional ways, the proposed method depends only on gene expression data. Therefore, it would be necessary to integrate other datasets, e.g., pathways, gene interactions, and genomic variants as future work. In addition, as our method is not able to produce a deterministic result, we would have to consider more precise tuning of our model and its parameters.

## Methods

### Ensemble *L*_*1*_-norm support vector machine

Support vector machine (SVM) has been an effective and popular method in machine learning, including the application of this method to biomedical problems [21]. Conventionally, SVM has been used for classification task, but it can be also applied to feature selection by considering the weights of the classifier [3]. In addition, instead of using general *L*_*2*_-norm for SVM, applying *L*_*1*_-norm which tends to produce sparse solutions, makes it possible to considerably reduce the number of features of a large feature set [22, 23]. The maximum number of features selected by *L*_*1*_-norm SVM is bounded by the number of samples [24]. Thus, in particular, it is suitable for biomarker selection from RNA-seq data in our study, which usually contains far more features than the number of samples. However, applying *L*_*1*_-norm SVM to a single dataset might produce a result that is excessively dependent on the sample set, which even causes uncertainty of reproducibility on datasets that are only slightly different. Moreover, the *L*_*1*_-norm is known to have difficulties in selecting closely correlated factors, as it tends to select only a single feature from among them and ignore the rest [25]. These problems can be addressed by applying *L*_*1*_-norm SVM to a perturbed dataset. Here, we propose the ensemble *L*_*1*_-norm SVM feature selection, combined with data perturbation to consider stability.

The flow of our stable *L*_*1*_-norm SVM algorithm can be described as follows:

1. Generate *n* random bootstrap samples, *X*_1_ *, X*_2_ *, …, X*_*n*_, using *i%* of data from the training dataset *X*.
2. Perform the cross-validation test on each bootstrap sample for setting regularization parameter *C*.
3. Apply *L*_*1*_-norm SVM to each bootstrap sample. Then the weight vector *w* is calculated for each feature.
4. Eliminate features of which the coefficient *w = 0* in each bootstrap sample.
5. Record the cross-validation score for each bootstrap sample for step (7).
6. Repeat step (2) ~ (5) until no more features of *w = 0* available for any bootstrap.
7. Select the optimal feature subsets for each bootstrap sample, which maximize the cross-validation score recorded in step (5).
8. Produce the integrated feature set of size *k* by aggregating all the remaining features in the bootstrap samples.
9. Convert *X* to the reduced dataset *X’* that consists of features in *k*. Here, the number of bootstrap samples that selected the feature is considered as ‘stability score’ *S* for each feature (1 ≤ *S* ≤ *n*).

For stable feature selection, we use bagging to generate *n* bootstrap samples from the training dataset [26, 27]. There has been no solid rule for the optimal value of *n*, and the related study shows that adjusting *n* only marginally affects the classification performance or stability [6]. Hence, *n* can be set moderately, though it is expected that slightly more converged result can be acquired by using larger value. After generating *n* bootstrap samples, the regularization parameter of *L*_*1*_-norm SVM is optimized for each of them. Unlike *L*_*2*_-norm SVM, the number of features to be reduced is automatically selected by the regularization parameter. The optimization problem of *L*_*1*_-norm SVM could be described as follows:

1 $$ \underset{w,b}{ \min}\left|\right|w\left|\right|{}_1+C{\displaystyle \sum_{i=1}^n \max {\left(0,\kern0.24em 1-{y}_i\left({w}^T{x}_i+b\right)\right)}^2}. $$

Here, parameter *C* reflects a regularization parameter that solves the trade-off problem between the training error and the complexity of the model and ||*w*||_1_ denotes the *L*_*1*_-norm of the weight vector *w*. We apply a linear kernel as a kernel function, as defined by

2 $$ k\left(x,y\right)={x}^Ty. $$

If the number of features is large, the linear kernel is efficient because mapping data to high-dimensional space usually does not improve the performance [28]. Hence, it is possible to acquire a comparable result at a much lower cost. In addition, the linear kernel is less prone to over-fitting than non-linear kernels, and the only parameter that needs to be optimized is *C*. We find the optimal value of *C* by applying a grid search using 10-fold cross-validation on the training dataset.

Next, *L*_*1*_-norm SVM is performed on each bootstrap sample to derive the sparse feature sets. In this step, the ranking criteria of SVM for each feature, i.e., the size of the weight vector *w* is not taken into account; for all the features of the coefficients *w* > 0, which means that features that are selected by at least one bootstrap sample are considered. After eliminating features whose coefficients are 0, we calculate and record the cross-validation score for each bootstrap sample. Similar to SVM-RFE, these processes above are repeated until no more features could be reduced by *L*_*1*_-norm SVM. However, reducing features does not always yield improved classification performance; sometimes the classification performance might decrease by excessive filtering of features. Thus, we select the feature subsets that maximize the cross-validation scores as optimal features for each bootstrap sample.

Then, the features that remain in all the bootstrap samples are aggregated. In the aggregation step, we only consider the frequency of each feature in whole bootstraps samples. It is plausible that the features remaining in more number of bootstrap samples are possibly more stable. Therefore, for each feature, we regard the number of bootstrap samples that selected the feature as its ‘stability score’. This score is valuable for regularization of the number of features, e.g., ignore those features selected from less than only 10% of bootstrap samples when there is a need to reduce the features to less than the number of samples, which commonly occurs in the biomedical field. In this study, instead of setting a specific cutoff point, we apply backward feature elimination based on the stability score to select the optimal subset, which is described in the next section. Finally, the dimension of the training dataset is reduced to the number of features that remain after the previous steps. As we have used the implementation of liblinear [29] for the feature selection, the time complexity of the algorithm can be described as follows O(n_f_ · n_b_), where n_f_ and n_b_ denote the number of features and bootstrap samples, respectively.

### Backward feature elimination for optimal feature subset selection

A stable *L*_*1*_-norm SVM provides an efficient solution for eliminating and selecting features. However, there might be a subset of which the performance exceeds that provided by using all the selected features. Therefore, an additional process capable of extracting the particular feature subset able to improve the classification performance is required. We utilize our stability score described in the previous section, the number of bootstrap samples that selected the feature. The stability score is aggregated from multiple bootstrap samples, thereby managing the stability issue that can be caused when using only a single dataset. We apply backward feature elimination to find one optimal subset of features of which the classification performance is best. We use SVM as classifier in the experiment, but other classifiers can also be applied. Here, as the number of features decreased considerably as a result of previous feature selection step, we apply the RBF kernel *k(x, y),* which generally performs better than the linear kernel.

3 $$ k\left(x,y\right)= \exp \left(-\gamma \left|x-y\right|{}^2\right). $$

Again, a grid search using 10-fold cross-validation was applied for optimizing the parameters of the RBF kernel, *C* and *γ*. The classification score is calculated on each fold of the cross-validation test and then aggregated. By eliminating the feature of which the stability score is lowest one by one, the classification score is calculated on all shrunk subsets until the size of the subset reaches 1. Finally, the one feature subset that succeeds in maximizing the classification performance over the cross-validation test is acquired. As the feature subset consists of a certain number of features, we do not need an additional process for setting the cutoff.

### Performance evaluation

Conventionally, the performance of feature selection has been evaluated by measuring the classification performance. However, the stability of selected features, which refers to the reliability and reproducibility of features, has also become a very important point recently. Instability of feature selection is mainly caused by a large number of features associated with small samples in data that complicate the proper reduction of features. These characteristics are common for biomedical data such as RNA-seq; therefore, a biomarker discovery study should consider stability as well as classification performance.

Hence, we evaluated the performance of our method by measuring the classification performance and their stability. Because our feature selection procedure contains a resampling step, we employ an independent training and test set in addition to the cross-validation test. We applied a well-known classification algorithm after feature selection to evaluate the classification performance. Statistical measures in the form of the accuracy, F1 score, Matthews correlation coefficient (MCC), and area under the curve (AUC) were measured together to evaluate the classification performance, as follows:

4 $$ Accuracy=\frac{TP+TN}{TP+TN+FP+FN,} $$

5 $$ F 1=\frac{2TP}{2TP+FP+FN,} $$

6 $$ MCC=\frac{\left(TP\cdot TN\right)-\left(FP\cdot FN\right)}{\sqrt{\left(TP+FP\right)\left(TP+FN\right)\left(TN+FP\right)\left(TN+FN\right)},} $$

7 $$ AUC={\displaystyle {\int}_0^1 ROC(x)}dx. $$

Here, TP, TN, FP, FN, and ROC represent true positive, true negative, false positive, false negative, and receiver operating characteristic curve, respectively.

Then we tested the stability of selected features by calculating the Tanimoto distance that has been applied in several previous studies [30, 31]. The Tanimoto distance is a statistical measure for calculating overlaps between two sets of elements of arbitrary cardinality. The Tanimoto distance *T* is calculated as follows:

8 $$ T\left({S}_i,{S}_j\right)=1-\frac{\left|{S}_i\left|+\right|{S}_j\left|-2\right|{S}_i\cap {S}_j\right|}{\left|{S}_i\right|+\left|{S}_j\right|-\left|{S}_i\cap {S}_j\right|,} $$

where |*S*_*i*_| and |*S*_*j*_| denote the number of elements in sets *S*_*i*_ and *S*_*j*_, respectively. Here, the Tanimoto distance *T*_*n*_ is obtained over multiple *n* sets of samples by calculating the arithmetic mean of *T* for each set of pairs, as described below.

9 $$ {T}_n\left({S}_i,{S}_j\right)=\frac{2}{n\left(n-1\right)}{\displaystyle \sum_{i=1}^{n-1}{\displaystyle \sum_{j=2}^n\left(T\left({S}_i,{S}_j\right)\right).}} $$

The Tanimoto distance takes values between 0 and 1, where 0 means that there is no overlap between the two sets, and 1 that the two sets have identical elements.

## References

[1] He Z, Yu W (2010). Stable feature selection for biomarker discovery. Comput Biol Chem.

[2] Saeys Y, Inza I, Larrañaga P (2007). A review of feature selection techniques in bioinformatics. Bioinformatics.

[3] Guyon I, Elisseeff A (2003). An introduction to variable and feature selection. J Mach Learn Res.

[4] Hall MA (1999). Correlation-based feature selection for machine learning.

[5] Saeys Y, Abeel T, Van de Peer Y. Robust feature selection using ensemble feature selection techniques. Joint European Conference on Machine Learning and Knowledge Discovery in Databases. Springer; 2008. p. 313–325. http://link.springer.com/chapter/10.1007%2F978-3-540-87481-2_21.

[6] Abeel T, Helleputte T, Van de Peer Y, Dupont P, Saeys Y (2010). Robust biomarker identification for cancer diagnosis with ensemble feature selection methods. Bioinformatics.

[7] Dernoncourt D, Hanczar B, Zucker J-D (2014). Stability of ensemble feature selection on high-dimension and Low-sample size data-influence of the aggregation method. ICPRAM.

[8] Bach FR. Bolasso: model consistent lasso estimation through the bootstrap. In Proceedings of the 25th international conference on Machine learning: 2008. ACM: 33–40.

[9] Meinshausen N, Bühlmann P (2010). Stability selection. J R Stat Soc Ser B Stat Methodol.

[10] Yu L, Liu H (2003). Feature selection for high-dimensional data: a fast correlation-based filter solution. ICML.

[11] Díaz-Uriarte R, De Andres SA (2006). Gene selection and classification of microarray data using random forest. BMC Bioinformatics.

[12] Guyon I, Weston J, Barnhill S, Vapnik V (2002). Gene selection for cancer classification using support vector machines. Mach Learn.

[13] Broad Institute TCGA Genome Data Analysis Center: Broad Institute of MIT and Harvard; 2015

[14] Li B, Dewey CN (2011). RSEM: accurate transcript quantification from RNA-Seq data with or without a reference genome. BMC Bioinformatics.

[15] Guinan P, Sobin LH, Algaba F, Badellino F, Kameyama S, MacLennan G, Novick A (1997). TNM staging of renal cell carcinoma. Cancer.

[16] Braga-Neto UM, Dougherty ER (2004). Is cross-validation valid for small-sample microarray classification?. Bioinformatics.

[17] Hall M, Frank E, Holmes G, Pfahringer B, Reutemann P, Witten IH (2009). The WEKA data mining software: an update. ACM SIGKDD Explorations Newsletter.

[18] Pedregosa F, Varoquaux G, Gramfort A, Michel V, Thirion B, Grisel O, Blondel M, Prettenhofer P, Weiss R, Dubourg V (2011). Scikit-learn: machine learning in python. J Mach Learn Res.

[19] Jagga Z, Gupta D. Classification models for clear cell renal carcinoma stage progression, based on tumor RNAseq expression trained supervised machine learning algorithms. In BMC proceedings: 2014. BioMed Central Ltd: S2. 10.1186/1753-6561-8-S6-S2 PMC4202178 25374611

[20] Haury A-C, Gestraud P, Vert J-P (2011). The influence of feature selection methods on accuracy, stability and interpretability of molecular signatures. PLoS One.

[21] Noble WS (2006). What is a support vector machine?. Nat Biotechnol.

[22] Bradley PS, Mangasarian OL (1998). Feature selection via concave minimization and support vector machines. ICML.

[23] Zhu J, Rosset S, Hastie T, Tibshirani R (2004). 1-norm support vector machines. Adv Neural Inf Proces Syst.

[24] Efron B, Hastie T, Johnstone I, Tibshirani R (2004). Least angle regression. Ann Stat.

[25] Friedman J, Hastie T, Tibshirani R (2010). Regularization paths for generalized linear models via coordinate descent. J Stat Softw.

[26] Efron B, Tibshirani RJ. An introduction to the bootstrap. CRC press; 1994. https://www.crcpress.com/An-Introduction-to-the-Bootstrap/Efron-Tibshirani/p/book/9780412042317.

[27] Breiman L (1996). Bagging predictors. Mach Learn.

[28] Hsu C-W, Chang C-C, Lin C-J (2003). A practical guide to support vector classification.

[29] Fan R-E, Chang K-W, Hsieh C-J, Wang X-R, Lin C-J (2008). LIBLINEAR: a library for large linear classification. J Mach Learn Res.

[30] Kalousis A, Prados J, Hilario M (2007). Stability of feature selection algorithms: a study on high-dimensional spaces. Knowl Inf Syst.

[31] Jiang Z, Xu R (2015). A novel feature extraction approach for microarray data based on multi-algorithm fusion. Bioinformation.

